# Evidence for a specific host-endosymbiont relationship between ‘*Rickettsia* sp. genotype RF2125’ and *Ctenocephalides felis orientis* infesting dogs in India

**DOI:** 10.1186/s13071-015-0781-x

**Published:** 2015-03-23

**Authors:** Sze-Fui Hii, Andrea L Lawrence, Leigh Cuttell, Rebecca Tynas, Puteri Azaziah Megat Abd Rani, Jan Šlapeta, Rebecca J Traub

**Affiliations:** School of Veterinary Science, The University of Queensland, Gatton, Queensland 4343 Australia; Faculty of Veterinary Science, The University of Sydney, New South Wales, 2006 Australia; Safe Food Production Queensland, PO Box 440, Spring Hill, Queensland 4004 Australia; School of Medicine and Pharmacology, The University of Western Australia, Nedlands, Western Australia 6009 Australia; Faculty of Veterinary Medicine, Universiti Putra Malaysia, 43400 Serdang, Selangor Malaysia; Faculty of Veterinary and Agricultural Sciences, The University of Melbourne, Parkville, Victoria 3052 Australia

**Keywords:** *Rickettsia* sp. genotype RF2125, Flea, Siphonaptera, India, Coevolution, Dogs

## Abstract

**Background:**

Fleas of the genus *Ctenocephalides* serve as vectors for a number of rickettsial zoonoses, including *Rickettsia felis*. There are currently no published reports of the presence and distribution of *R. felis* in India, however, the ubiquitous distribution of its vector *Ctenocephalides felis*, makes it possible that the pathogen is endemic to the region. This study investigates the occurrence of *Rickettsia* spp. infection in various subspecies of *C. felis* infesting dogs from urban areas of Mumbai, Delhi and Rajasthan in India.

**Methods:**

Individual fleas collected off 77 stray dogs from Mumbai, Delhi and Rajasthan were screened for *Rickettsia* spp. by a conventional PCR targeting the *omp*B gene. Further genetic characterisation of *Rickettsia*-positive fleas was carried out using nested PCR and phylogenetic analysis of partial DNA sequences of the *glt*A and *omp*A genes. *Ctenocephalides* spp. were morphologically and genetically identified by PCR targeting a fragment of *cox*1 gene.

**Results:**

Overall, 56/77 fleas (72.7%), including 22/24 (91.7%) from Delhi, 32/44 (72.7%) from Mumbai and 2/9 (22.2%) from Rajasthan were positive for *Rickettsia* DNA at the *omp*B gene. Sequences of *glt*A fragments confirmed the amplification of *Rickettsia* sp. genotype RF2125. The *omp*A gene of *Rickettsia* sp. genotype RF2125 was characterised for the first time and shown 96% identical to *R. felis*. Three species of *Ctenocephalides* were identified, with the *Ctenocephalides felis orientis* being the dominant flea species (69/77; 89.6%) in India, followed by *Ctenocephalides felis felis* (8/77; 10.4%).

**Conclusions:**

High occurrence of *Rickettsia* sp. genotype RF2125 in *C. felis orientis* and the absence of *R. felis* suggests a specific vector-endosymbiont adaptation and coevolution of the *Rickettsia felis-like* sp. within subspecies of *C. felis*.

## Background

Rickettsioses caused by *Rickettsia* spp. are zoonotic vector-borne diseases that have a cosmopolitan distribution. In India, infection with epidemic typhus caused by *Rickettsia prowazekii* [1], scrub typhus caused by *Orientia tsutsugamushi* [2,3], murine typhus caused by *Rickettsia typhi* [4], Mediterranean spotted fever caused by *Rickettsia conorii* [5,6] and infection by *Candidatus Rickettsia kellyi* [7] have been reported in humans. Clinical signs in humans typically manifest as febrile illness with myalgia, headache, enlarged painful lymph nodes, a cutaneous ‘rash’, eschar (necrosis at the bite site), respiratory, gastrointestinal and/ or neurological abnormalities [7-9].

In recent years, the ubiquitous nature and public health significance of *Rickettsia felis*, an emerging rickettsial zoonosis that causes flea-borne spotted fever (FSF) has become increasingly apparent. To date, over 100 human cases have been reported worldwide including in Europe, the Americas, the United States of America (USA), Southeast Asia, Africa and the Middle East [10]. The cat flea, *Ctenocephalides felis*, is the recognised biological vector and infections of *R. felis* have been reported in over 25 countries spanning five continents, with infection rates ranging from 15% in New Zealand to 81% in New Caledonia [11,12]. More recently, domestic dogs have also been identified as potential natural mammalian reservoirs for *R. felis* [13,14]. There are currently no published reports of the presence and distribution of *R. felis* in India, however, its ubiquitous distribution makes it likely that the pathogen is also endemic to the region.

In India, both flea vectors and canine reservoirs live in close proximity to humans in rural and urban communities. India is estimated to have a stray dog population of 25 million [15] and a pet dog population of over 10 million [16]. Visual inspection of stray dogs from urban areas of Delhi, Mumbai and Sikkim reported a prevalence of flea infestation 40.7%, 42.6% and 75.2% respectively [17]. In Rajasthan, 6% of dogs were reported visually infested with fleas (data not shown). Although human infection with *R. felis* has not been reported in India, it is possible that the non-specific symptoms that mimic other rickettsial or viral infections coupled with the low clinical index of suspicion for FSF, and low availability of specific diagnostic tests such as PCR, culture and *R. felis*-specific serological tests, make it likely that many human cases are grossly misdiagnosed.

In the present study, we aim to genetically identify and determine the prevalence of *Rickettsia* spp. in various subspecies of *Ctenocephalides* spp. collected from stray dogs in urban areas of Delhi, Mumbai and Rajasthan. Morphology and molecular genotyping based on the mtDNA cytochrome c oxidase subunit I (*cox*1) gene was applied to demonstrate presence of *Ctenocephalides felis felis* and *Ctenocephalides felis orientis* and *Ctenocephalides canis*.

## Methods

### Flea collection

Fleas were collected by flea combing the coat of 324 stray dogs undergoing sterilisation through animal birth control and rabies vaccination programs in Mumbai (n = 162), Delhi (n = 162) and Rajasthan (n = 150). For further details of methods and prevalence of flea infestation in dogs for Mumbai and Delhi sampling sites, refer to Abd Rani (2011) [18]. All fleas were fixed in 70% ethanol and transported to the University of Queensland and the University of Melbourne for analysis. A total of 77 fleas, each randomly collected from individual stray dogs in the city of Mumbai (n = 44) and Gurgaon in Delhi (n = 24), and all dogs in Jaipur in Rajasthan (n = 9) were selected for identification and *Rickettsia* spp. screening using PCR. A single *C. canis* and two *C. felis felis* voucher specimens fixed in 70% ethanol were sourced from dogs in the Sikkim area, northeast India. Ectoparasite sampling in Delhi and Mumbai was approved by the University of Queensland Animal Ethics Committee. In Rajasthan, ectoparasite sampling was carried out in accordance with the Animal Welfare Act (2011) of India and overseen by Dr Jack Reece, Veterinarian-in-Charge, Help In Suffering, Rajasthan, India.

### Flea identification and extraction of DNA

From selected voucher flea species, total DNA was extracted from fleas whilst retaining flea exoskeletons [19,20]. DNA was isolated using Isolate II Genomic DNA kit (BioLine, Australia) as previously described [20]. DNA was eluted into 50 μL of Tris buffer (pH = 8.5) and stored at −20°C. The flea exoskeleton was soaked in 10% KOH for approximately an hour. Exoskeletons were dehydrated using a series of ethanol washes (70%, 80%, 95%, absolute) for 1 hour each, and slide-mounted in Euparal (Ento Supplies, Australia). The slides were donated to the Australian National Insect Collection (ANIC) in Canberra, Australia. Fleas were identified morphologically using a compound microscope with the aid of keys and descriptions [21,22].

Seventy-seven individual fleas were rinsed with PBS for 10 minutes and mechanically crushed using pellet pestles in a 1.5 ml microcentrifuge tube. Genomic DNA was extracted using the DNeasy Blood & Tissue Kit® (Qiagen, Hilden, Germany) according to the manufacturer’s instructions and eluted in 50 μl of AE Buffer. These samples were then subjected to molecular identification using direct sequence comparisons to those deposited on GenBank and screened for *Rickettsia* spp. using PCR.

### Amplification and phylogenetic analysis of the mtDNA cytochrome c oxidase subunit 1 of fleas

A 5′ fragment of the cytochrome c oxidase subunit I (*cox*1) coding for COX1 protein was PCR amplified using the generic invertebrate amplification primers: LCO1490 (5′-GGT CAA CAA ATC ATA AAG ATA TTG G-3′)/HC02198 (5′-TAA ACT TCA GGG TGA CCA AAA AAT CA-3′) [23] and Cff-F [S0367] (5′-AGA ATT AGG TCA ACC AGG A-3′) and Cff-R [S0368] (5′-GAA GGG TCA AAG AAT GAT GT-3′) [20] or their combination as well as MLepF1 (5′-GCT TTC CCA CGA ATA AAT AAT A-3′) [24] and HC02198 (5′-TAA ACT TCA GGG TGA CCA AAA AAT CA-3′). Reactions of 30 μl contained MyTaq Red Mix (BioLine, Australia), and approximately 1–10 ng of genomic DNA template (~2 μl). Alternatively, 25 μl reactions contained 5× PCR buffer, 200 μmol dNTP, 1.5 mmol MgCl_2_, 0.5 units of GoTaq polymerase (Promega). Primers were added at a final concentration of 10 pmol. The cycling was as follows (BioLine mix): denaturing at 95°C for 1 min followed by 35 cycles of 95°C for 15 s, 55°C for 15 s, 72°C for 10 s, and a final elongation for 5 min at 72°C. For the alternative PCR, the cycling was as follows (Promega mix): denaturing at 95°C for 2 min followed by 35 cycles of amplification at 95°C for 30 s, 55°C for 30 s and 72°C for 30 s, and a final extension step of 72°C for 5 min. All PCRs were run with a negative control of sterile PCR-grade water. A positive control with flea DNA representing each of species/subspecies morphologically identified and known to amplify at these conditions from a previous study was included in each run [20].

Aliquots of all PCR reactions were subjected to agarose gel electrophoresis to verify product size and the remainder was submitted for sequencing (Macrogen Ltd, Seoul, Korea). The sequences of voucher flea species have been deposited in GenBank (GenBank: KP229378-KP229385).

Individual sequences of the voucher flea specimens were assembled with CLC Main Workbench 6.9 (CLCbio, Denmark). Composition of the nucleotide sequences and phylogenetic analysis were determined using MEGA6.06 [25]. Sequence divergences were calculated using the Kimura 2 parameter distance model.

### Amplification of the *omp*B, *glt*A and *omp*A genes of *Rickettsia* spp.

Individual flea DNA was initially screened for spotted-fever group *Rickettsia* spp. with previously described conventional PCR targeting a 297-bp region of the rickettsial outer membrane protein B (*omp*B) gene [13,26]. Randomly selected *Rickettsia*-positive fleas were further characterised with conventional nested PCRs on more variable loci targeting a 654 bp fragment of *glt*A and a 879 bp fragment of the *omp*A genes of *R. felis* [14,27]. Secondary *omp*A primers comprising *omp*A-F2 (5′-CGGTACAATCATTGCAACTGG-3′) and *omp*A-R2 (5′-GCTATATCTTCAGCAAATAACG-3′) were designed to increase the sensitivity of the PCR by amplification of product from the primary round. PCR conditions of the secondary PCR were identical to that of the primary [27]. To prevent cross-contamination of DNA, DNA extraction, PCR setup, DNA loading for secondary nested PCR and detection of amplicons were carried out in separate laboratories. Negative control using nuclease-free water was included in every PCR run.

Positive PCR products were submitted for DNA sequencing. DNA sequences were analysed using Finch TV 1.4.0 (Geospiza Inc.) and compared with those available in GenBank using the BLAST algorithm (BLAST Basic Local Alignment Search Tool, 2014). DNA sequences were aligned using BioEdit version 7.2.3 [28] with previous published sequences of the *glt*A and *omp*A gene of various rickettsiae species sourced from GenBank. Neighbor-joining analyses were conducted with Tamura-Nei parameter distance estimates, and trees constructed using Mega 4.1 software (www.megasoftware.net). Bootstrap analyses were conducted using 1000 replicates. The sequences of both *glt*A and *omp*A genes of *Rickettsia* spp. have been deposited in GenBank (accession no. KP256357-KP256359, KP406620-KP40662, KP687803-KP687805).

### Statistical methods

A Fisher’s Exact Test was performed to determine whether an association exists between the proportions of *Rickettsia* spp*.* infection among different subspecies of *C. felis* identified on surveyed dogs using Vassarstats (http://vassarstats.net/tab2x2.html). Odds ratios were calculated to describe the strength of the association.

## Results

Overall, 56/77 fleas (72.7%), including 22/24 (91.7%) from Delhi, 32/44 (72.7%) from Mumbai and 2/9 (22.2%) from Rajasthan were positive for *Rickettsia* spp. at the *omp*B gene. All negative controls in each PCR run were PCR-negative. Direct alignment of the partial *omp*B sequences of *Rickettsia* isolated from all Indian fleas revealed 99.6% similarity to validated *R. felis* isolate URRWXCal2 (GenBank: CP000053).

**Figure 1:**
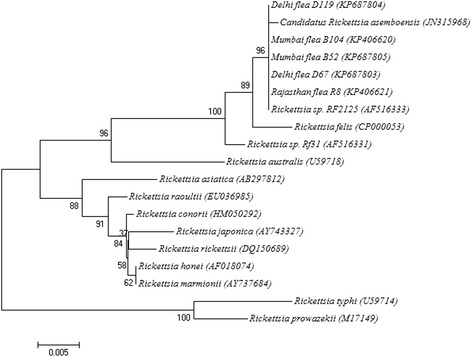
**Neighbor-joining analysis based on the alignment of the partial** ***glt*** **A gene of rickettesiae.**

Forty-six (12 Delhi, 32 Mumbai and 2 Rajasthan) and 25 (12 Delhi, 11 Mumbai and 2 Rajasthan) fleas that were positive for *Rickettsia* at the *omp*B gene were subjected to further PCRs targeting the more variable *glt*A and *omp*A genes, respectively. The sequences of the *glt*A fragments identified in 21 *C. felis* isolates were 100% identical to each other and to *Rickettsia* sp. genotype RF2125 (GenBank: AF516333) and 99.8% identical to *Candidatus* Rickettsia asemboensis (GenBank: JN315968). Neighbour joining analysis based on the alignment of partial *glt*A sequence provided strong bootstrap support for the placement of five randomly selected representatives of *Rickettsia* isolated from *C. felis* into the same cluster as *Rickettsia* sp. genotype RF2125 (GenBank: AF516333) and *Candidatus* Rickettsia asemboensis (GenBank: JN315968) (Figure 1).

**Figure 2:**
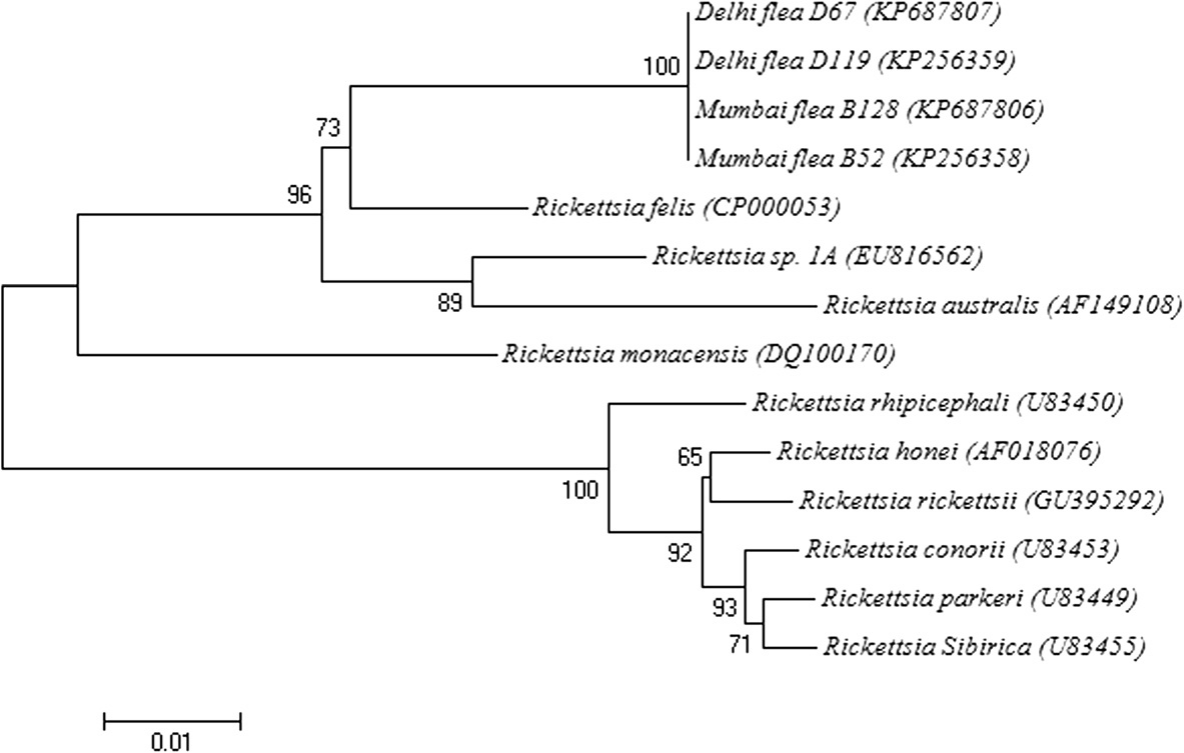
**Neighbor-joining analysis based on the alignment of the partial** ***omp*** **A gene of rickettesiae.**

Sequences of the *omp*A fragment amplified from 18 *C. felis* were 100% identical to each other and 96% identical to validated *R. felis* isolate URRWXCal2 (GenBank: CP000053). Phylogenetic analysis of the *omp*A gene revealed moderate support for the placement of all isolates of *Rickettsia* spp. from Indian fleas within a single cluster distinct to validated *R. felis* isolate URRWXCal2 (GenBank: CP000053) (Figure 2).

**Figure 3:**
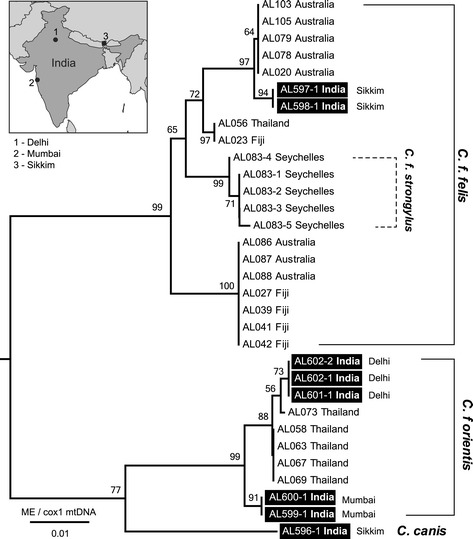
**Phylogenetic relationships of** ***Ctenocephalides felis*** **and** ***Ctenocephalides canis*** **based on nucleotide sequence of the mtDNA** ***cox*** **1.** The tree was inferred using the Minimum Evolution method with distances calculated using Kimura 2-parameter method. There were a total of 658 positions in the final dataset. For the tree shown, all ambiguous positions were removed for each sequence pair. The numbers above the branches indicate percentage of 1000 replicate trees in which the associated taxa clustered together in the bootstrap test. The tree is drawn to scale, with branch lengths in the same units as those of the evolutionary distances used to infer the phylogenetic tree. The tree was rooted using *Bradiopsylla echidnae* mtDNA *cox*1 sequence (not shown). The scale is in the units of the number of base substitutions per site. Evolutionary analyses were conducted in MEGA6. Flea species is shown on the right and terminal nodes are labelled with their unique identifier and country of origin. Fleas from India are in black boxes followed by the locality where it was collected, a map is shown in the inset.

**Figure 4:**
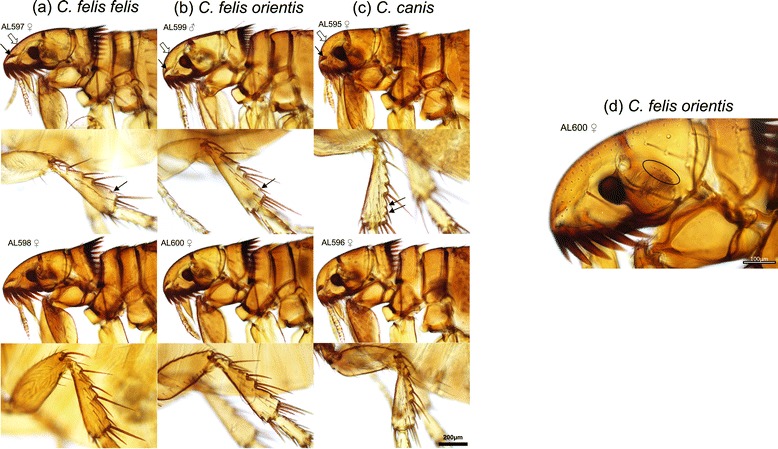
**Diagnostic morphological features for differentiation of** ***Ctenocephalides felis felis, Ctenocephalides felis orientis*** **and** ***Ctenocephalides canis*** **. (a)** *C. felis felis* is characterised by a long, acutely angled frons with no part on the vertical plane (white arrow). The dorsal incrassation is long and narrow (black arrow). The posterior margin of the hind tibia of this species has only one notch bearing a stout seta between the longer post-median and apical setae. **(b)** *C. felis orientis* is characterised by a short, rounded frons (white arrow) and a shorter dorsal incrassation compared to *C. felis felis* (black arrow). The posterior margin of the hind tibia is as seen in *C. felis felis* (black arrow). **(c)** *C. canis* is characterised by a short, sharply vertical frons (white arrow) and a short, club-shaped dorsal incrassation. The posterior margin of the hind tibia has two notches bearing stout setae between the post-median and apical setae (black arrows). **(d)** *C. felis orientis* can further be distinguished by the presence of a row of tiny setae just dorsal of the antennal fossa in the female (circled), numerous of which are seen in all males of the genus *Ctenocephalides*.

Phylogenetic analysis based on the *cox*1 fragment placed voucher *C. felis felis* specimens from Sikkim, India within *C. felis felis* and as a closely related group (two nucleotide polymorphisms across 513 nt) to *C. felis felis* haplotype 1 from Australia [20]. The sequences of voucher *C. felis orientis* strains from Delhi clustered within *cox*1 sequences from *C. felis orientis* from Thailand (Figure 3) and *cox1* sequences from voucher *C. felis orientis* strains from Mumbai formed a sister group *C. felis orientis*, that we consider *C. felis orientis.* All *C. felis orientis* from Mumbai and Delhi were morphologically consistent with descriptions of *C. felis orientis* (Figure 4)*. C. felis orientis* formed a sister group with *C. canis* collected on dogs from Sikkim (Figure 3).

*Ctenocephalides felis* species were morphologically identified infesting all dogs in Mumbai, Delhi and Rajasthan (Figures 3, 4). Within *C. felis*, two subspecies were recognised *C. felis orientis* and *C. felis felis* (Figure 4)*.* PCR targeting mtDNA *cox*1 gene sequence of *C. felis* using primer pair LCO1490/Cff-R was amplified in 8/77 fleas. The DNA sequences of all eight fleas showed 100% identity to *C. felis felis* haplotype 3 isolated from Thailand (GenBank: KF684866) and Fiji (GenBank: KF684877) and 98.8% identity to *C. felis felis* haplotype 1 from Sydney, Australia (KF684882). Fleas that failed to amplify on the first PCR were subjected to a second PCR using primer pair Cff-F/HC02198 that successfully amplified the remaining 69 fleas, of which 22 were subjected to DNA sequencing and identified as *C. felis orientis*. The dominant flea species infecting dogs in Mumbai, Delhi and Rajasthan was the Oriental cat flea (*C. felis orientis*) (Table 1).

**Table 1:** **Distribution of** ***Rickettsia*** **sp. genotype RF2125 in flea subspecies sampled at three different locations in India**

**Location**	***C. felis orientis***	***C. felis felis***	**Total**
Mumbai	31/37 (83.8%)	1/7 (14.3%)	32/44 (72.7%)
Delhi	22/24 (91.7%)	-	22/24 (91.7%)
Rajasthan	2/8 (25%)	0/1 (0%)	2/9 (22.2%)
**Total**	**55/69 (79.7%)**	**1/8 (12.5%)**	**56/77 (72.7%)**

Statistically, *C. felis orientis* fleas were 27.5 times more likely to be PCR-positive for ‘*Rickettsia* sp. genotype RF2125’ than *C. felis felis* (p = 0.00005).

## Discussion

To our knowledge, this is the first study to molecularly characterise rickettsial infection in fleas from India. Previously, *C. canis* were reported to harbour spotted fever group rickettsiae on the basis of seroconversion in guinea pigs [29]. Dogs are implicated as potential vertebrate reservoir hosts for a number of zoonotic *Rickettsia*, including *R. rickettsii*, *R. conorii* and *R. felis* [13,30]. The highly ubiquitous nature of fleas and their rickettsial endosymbiont isolated from stray dogs in the current study suggests that this *R. felis*-like organism (*Rickettsia* sp. genotype RF2125) may also use dogs as reservoir hosts and pose a potential zoonotic risk to humans. In the current study, a conventional PCR was used to screen the rickettsiae. The distribution of *Rickettsia* spp. infection may probe higher should a more sensitive molecular assay such as real-time PCR was used in the study.

Since the first detection of *R. felis* in cat fleas in 1990, this zoonotic rickettsial pathogen has been reported in all continents, except Antarctica. The ubiquitous characteristic of *R. felis* is associated with the cosmopolitan distribution of the *C. felis.* Curiously, *R. felis* was not detected in any of the fleas sourced from stray dogs in this study. The current study provides evidence for the occurrence of *Rickettsia* sp. genotype RF 2125 as the dominant rickettsiae carried by fleas infesting dogs, with *C. felis orientis* as the primary carrier. In all cases, the species of *Rickettsia* detected in fleas in the current study were found to be identical to *Rickettsia* sp. genotype RF2125, originally detected in a single *C. felis* (subspecies unknown) and two *C. canis* isolates near the Thai-Myanmar border [31]. *Rickettsia* sp. genotype RF2125 was later described in a variety of flea species spanning nine countries -four *Archaeopsylla erinacei* sourced from hedgehogs in Algeria [32] and two sourced from foxes in France [33]; 12 *C. canis* isolated from dogs in Gabon [33]; 12 *Echidnophaga gallinacea* isolated from five black rats in Egypt [34]; a single *Pulex irritans* sourced from a dog in Hungary [35]; 6/209 *C. felis* sourced from dogs and cats and from 56/57 rats in Malaysia [36,37]; 2 pools of *C. felis* from a zookeeper and a grizzly bear in the USA [38], 44/81 *C. felis* pools sourced from dogs and cats in Costa Rica [39]; and *C. felis* and *C. canis* sourced from dogs and cats in Uruguay [40]. The aforementioned studies did not provide detailed morphological or molecular identification of *Ctenocephalides* spp. to a subspecies level. *Ctenocephalides felis* is the most common flea in the world with *C. felis felis* the most widespread subspecies [20]. Other subspecies are more geographically restricted, for example, *C. felis damarensis* to south western Africa, *C. felis strongylus* to the Ethiopian zoogeographic region and *C. felis orientis* to Asia, [20]. *C. canis* (Curtis) is also widespread but encountered less frequently than *C. felis*. It has been reported in the USA [41], South America [42], North Africa [43], Europe [44] and Asia [45]. Studies conducted in Thailand [46], north-west Laos and Sabah, Malaysia [47] reported that *C. felis orientis* was the most common flea species infesting domestic dogs (73.3% - 86.2%), which is comparable to our finding (89.6%; 69/77).

A single study by Kernif et al. (2012) identified species of *C. felis* sourced from dogs in Laos to a subspecies level [47]. Rickettsial DNA was detected in 69 of 90 (76.6%) fleas. All fleas positive for rickettsial DNA were positive by *R. felis*-specific qPCR targeting the *glt*A gene however it is unclear whether conventional PCR and DNA sequencing was carried out on these isolates to confirm their identity as *R. felis* URRWXCal2. With a mere two base pair difference, it is possible that the qPCR may have cross reacted with *Rickettsia* sp. genotype RF2125. Interestingly, Kernif et al. (2012) also discovered the frequency of ‘*R. felis’* significantly higher in *C. felis orientis* (59/66; 89.4%) than in *C. felis felis* (10/19; 52.6%) [47]. The association of the subspecies of cat flea to the species of *R. felis-*like rickettsiae may be attributed to host-endosymbiont coevolution. A significantly higher prevalence of *Rickettsia* sp. genotype RF2125 in *C. felis orientis* compared to *C. felis felis* in this study suggests that this species of flea could be the primary invertebrate reservoir in India and possibly other parts of Asia where *C. felis orientis* and *R. felis* sp. genotype RF2125 co-exist. In addition, in Africa [48] and Europe [49], between 95-100% of hedgehog fleas *A. erinacei* has been demonstrated as carriers of *R. felis*. The potential for this flea to also harbour *Rickettsia* sp. genotype 2125 indicates the potential for hedgehog fleas to act as additional vectors for *Rickettsia* sp. genotype RF2125. Nevertheless, the absence of *R. felis* in *C. felis orientis* sourced from Indian dogs and the absence of *R. felis* sp. genotype RF2125 from *C. felis* isolated from Australia, where only *C. felis felis* is known to occur, raises questions with regard to vector-endosymbiont adaptation and coevolution of the *Rickettsia felis-like* sp. within subspecies of *C. felis*.

Genetically, *C. felis orientis* is more closely related to the dog flea *C. canis. C. felis orientis* forms a sister group to *C. canis* (Figure 3) that is phylogenetically distinct to *C. felis. Rickettsia* sp. genotype RF2125 has been reported in the USA, Central and South America, North Africa and Europe, areas in which *C. felis orientis* is absent. Given that 12/12 *C. canis* collected from dogs in Gabon [33] were infected with *R. felis* sp. genotype RF2125, a co-evolutionary relationship between *Rickettsia* sp. genotype RF2125 and fleas belonging to the *C. canis/C. felis orientis* complex is likely and should be explored further*.*

In addition to characterisation at *omp*B and *glt*A gene fragments, a partial region of the *omp*A gene of *R. felis* sp. genotype RF2125 was characterised for the first time using published primers designed to be specific to *R. felis* URRWXCal2 [27]. *omp*A sequence of this amplified *Rickettsia* spp. was 96% identical to *R. felis* URRWXCal2, supporting its potential placement as a new species of *Rickettsia* [50]. Further demonstration of entire length sequences of other genes such as 16S rRNA and gene D is also required to classify *Rickettsia* sp. genotype RF2125 as new species [51]. Even though *Rickettsia* sp. genotype RF2125 has been genetically identified on multiple occasions since 2004, the species has never been isolated in cell culture. Nevertheless, a tentative species should be assigned as a matter of priority.

## Conclusion

In conclusion, our study provided the first insight of occurrence of *Rickettsia* sp. genotype RF2125 infection and its close association with *C. felis orientis*, the predominant ‘cat flea’ infesting dogs in India. Surveys that include detailed morphological and molecular characterisation of fleas together with their *R. felis*-like rickettsiae will shed further light on whether host-endosymbiont adaptation is observed in other regions of the world. It is unknown if *Rickettsia* sp. genotype RF2125 is pathogenic to humans. Nevertheless, this study reveals that the public are at a high risk of exposure to *R. felis* sp. genotype RF2125 through the bite of *C. felis orientis* fleas that are ubiquitous on dogs in India.

## Abbreviations

FSF: Flea-borne spotted fever
qPCR: Real-time polymerase chain reaction
